# Conserved Structural Features of Core Oligosaccharides among the Lipopolysaccharides of Respiratory Pathogens from the Genus *Bordetella* Analyzed Exclusively by NMR Spectroscopy

**DOI:** 10.3390/ijms22031029

**Published:** 2021-01-21

**Authors:** Karolina Ucieklak, Sabina Koj, Tomasz Niedziela

**Affiliations:** Hirszfeld Institute of Immunology and Experimental Therapy, 53-114 Wroclaw, Poland; karolina.ucieklak@hirszfeld.pl (K.U.); sabina.koj@hirszfeld.pl (S.K.)

**Keywords:** *Bordetella* spp., lipopolysaccharide, core oligosaccharide, NMR spectroscopy, whooping cough, pertussis

## Abstract

Bacterial pathogens expose on the cell surface a variety of complex carbohydrate molecules. Gram-negative bacteria produce lipopolysaccharides, which are the main components of the outer membrane of bacterial envelopes and play a major role in host–pathogen interactions. *B. pertussis*, *B. parapertussis*, *B. bronchiseptica,* and *B. holmesii*, are mammalian respiratory pathogens, having substantial economic impact on human health and agriculture. *B. pertussis* is responsible for whooping cough (pertussis) and *B. holmesii* is the second pertussis etiological factor, but the current anti-pertussis vaccines do not provide cross-protection. The structural data on any given hypothetical carbohydrate antigen is a prerequisite for further analysis of structure-related activities and their interaction with hosts. ^1^H NMR spectra constitute fingerprints of the analyzed glycans and provide unique identity information. The concept of structure-reporter groups has now been augmented by ^1^H,^13^C-correlation spectra of the *Bordetella* oligosaccharides. The comparative analysis of *Bordetellae* oligosaccharides (OS) revealed that the hexasaccharide, comprising the α-Glc*p*N, α-Glc*p*A, 4,6-disubstituted-β-Glc*p*, 2,7-disubstituted-l-α-d-Hep*p*, 3,4-disubstituted-l-α-d-Hep*p,* and Kdo, constitute the least variable OS segment. This minimal common element in the structure of lipopolysaccharides of *Bordetellae* could be used to devise a universal cross-protective vaccine component against infections with various bacteria from the genus *Bordetella*.

## 1. Introduction

The genus *Bordetella* comprises a group of aerobic Gram-negative small coccobacilli. Most of these bacteria have adapted to live in a close relation with higher organisms. The four species of *Bordetella, B. pertussis*, *B. parapertussis, B. bronchiseptica,* and the lesser known *B. holmesii*, are respiratory pathogens of mammals, having substantial economic impact on human health and agriculture [1]. The classical *Bordetellae* comprises *B. pertussis*, *B. parapertussis,* and *B. bronchiseptica.* These bacteria are genetically related, often designated as the “*Bordetella bronchiseptica* cluster”, and share some structural features regarding their surface glycan antigens.

Among them, *B. pertussis* remains as an important human pathogen, responsible for whooping cough (pertussis), a highly contagious illness affecting the respiratory tract, which is especially severe for newborns and young children [2,3]. Even with the common availability of vaccination, the number of reported pertussis cases has been on the rise in recent years [4,5,6]. The main reason for the resurgence of this vaccine-preventable disease is a waning of vaccine-induced immunity and genetic changes in *B. pertussis* strains [7]. In recent years, *B. holmesii*, another human pathogen frequently isolated from immunocompromised patients, has also been implicated in a less severe pertussis-like illness. *B. holmesii* has become the second pertussis etiological factor, but the currently used anti-pertussis vaccines do not provide cross-protection [8]. An unequivocal discrimination between *B. pertussis* and *B. holmesii* and thus possible misdiagnosis can interfere with actual efficiency of the anti-pertussis vaccination.

Bacterial pathogens expose on the cell surface a variety of complex carbohydrate molecules that are essential for the structural integrity and interactions with hosts [9,10,11]. Bacteria produce several types of extracellular polysaccharides vital for colonization and pathogenesis. Gram-negative bacteria produce lipopolysaccharides (LPS), which are the main components of the outer membrane of bacterial envelopes. LPS are among the virulence determinants and the most abundant surface molecule of Gram-negative bacteria. LPS play a major role in the pathogen interactions with the immune system of the host and manifest endotoxic activities similar to those of enteric bacteria. LPS structures of *B. pertussis* are limited to two types, comprising a lipid A linked to a nonasaccharide core alone or extended by a distal trisaccharide. The LPS of *B. pertussis* lack the O-specific polysaccharide chain, thus structurally constituting a lipooligosaccharide (LOS) [12]. Other species from the genus *Bordetella* have more structurally diverse LPS, including variable O-specific polysaccharides, structurally conserved core oligosaccharides, and unique linking elements [13].

It is well known that protective human immunity to bacterial pathogens correlates with serum bactericidal activity of antibodies specific for cell surface carbohydrate antigens [14]. The carbohydrate component, such as a selected glycan segment of LPS or a capsular polysaccharide, can be converted into a non-toxic, highly immunogenic, T-dependent antigen by conjugation to a protein carrier [4,15,16]. In our previous studies such covalent conjugates of oligosaccharides with tetanus toxoid induced production of IgG antibodies against core oligosaccharides of *E. coli* in animal models. These antibodies reacted strongly with LPS in the presence of serum proteins and recognized LPS present on the surface of live bacteria [17,18]. Whereas antibodies produced against the components of the acellular pertussis vaccine, which is devoid of LOS, did not promote the complement-dependent killing of bacteria [19,20].

The complete structural data on any given hypothetical carbohydrate antigen is a prerequisite for further analysis of structure-related activities and interactions with hosts triggered by these molecules. ^1^H NMR spectra constitute fingerprints of the analyzed glycans, comprising detailed and unique identity information on their monosaccharide constituents and their mutual relationships. The concept of the structure-reporter groups [21] in the NMR spectra of complex glycans relies on the identification of the feature characteristics for distinct structural elements in carbohydrate structures, including (1) resonances in the anomeric region (number of resonances, their pattern), (2) protons attached to carbons at the linkage positions and in direct vicinity of such linkages, (3) protons at the deoxy-carbon atoms, (4) protons of *N*-acetyl and *N*-methyl groups as well as protons affected by phosphate substitutions. We have extended this concept in the analysis of core oligosaccharides of *Bordetellae* by application of the ^1^H,^13^C HSQC-DEPT spectra of the isolated oligosaccharides for direct comparison of the structural features, which could not be resolved using 1D profiles alone. This approach allowed us to describe, for the first time, the structures of the core oligosaccharides (OS) of *B. bronchiseptica* strains 530 and 1943, as well as *B. parapertussis* strain 529, indicating the minute variations among these oligosaccharides in relation to a typical LPS of the “*B. bronchiseptica* cluster”.

We now report on the application of a solely NMR approach to the identification of the least variable segment of the LPS core oligosaccharides among the selected species and strains of the genus *Bordetella*. As demonstrated before, the antibodies generated against the glycoconjugates of OS isolated from rough *E. coli* strains cross-recognized the core oligosaccharides among smooth strains [22]. Such a minimal common element in the structure of *Bordetellae* lipopolysaccharides could be used to devise a new pertussis vaccine component, capable of protecting against infections with various species of bacteria of the genus *Bordetella*.

## 2. Results

### 2.1. Isolation of Lipopolysaccharides and Core Oligosaccharides

The *Bordetellae* used in this study comprise three classical species, i.e., *B. pertussis* strains 186 and 606, *B. parapertussis* strain 529, *B. bronchiseptica* strains 530 and 1943, and an emerging *B. holmesii* ATCC 51541. All strains were cultured using the standard Steiner-Scholte medium. The lipopolysaccharides (LPS) were isolated by the modified hot phenol/water extraction method according to Westphal and Jann [23] and purified by ultracentrifugation.

**Figure 1:**
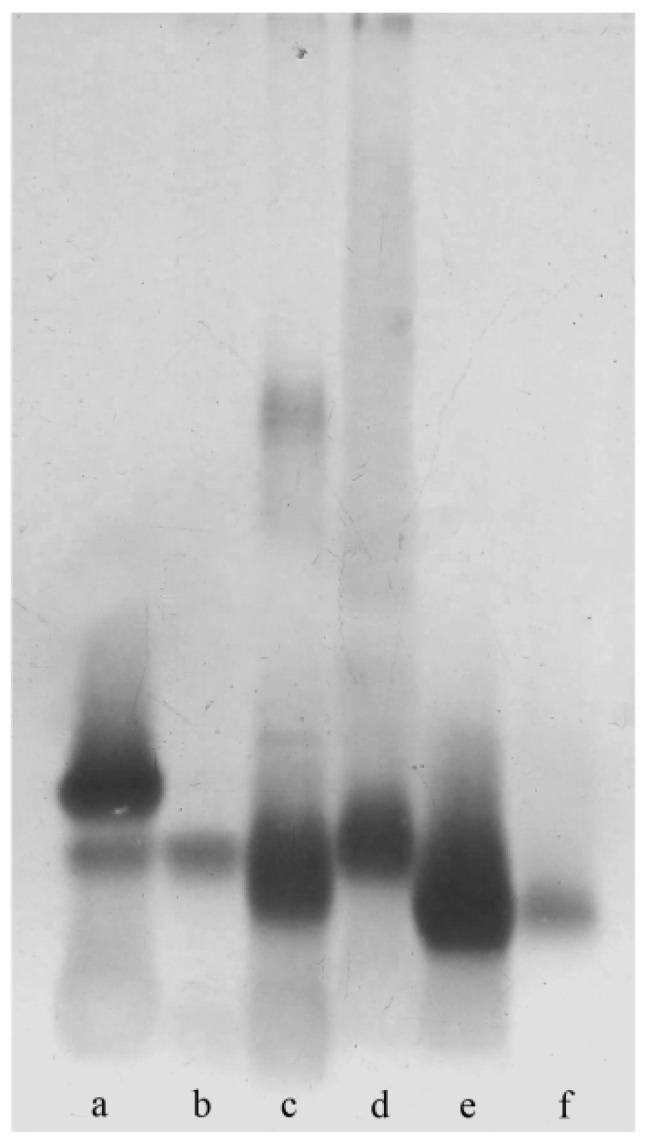
SDS-PAGE profiles of the lipopolysaccharides (LPS) isolated from (**a**) *B. pertussis* 186, (**b**) *B. pertussis* 606, (**c**) *B. parapertussis* 529, (**d**) *B. holmesii* ATCC 51541, (**e**) *B. bronchiseptica* 530, and (**f**) *B. bronchiseptica* 1943.

The SDS-PAGE analysis of *Bordetella* species LPS (Figure 1) revealed the smooth-type LPS for *B. parapertussis* 529 and *B. holmesii* ATCC 51541 and the rough-type LOS for *B. pertussis* strains 186 and 606 as well as for *B. bronchiseptica* strains 530 and 1943.

**Figure 2:**
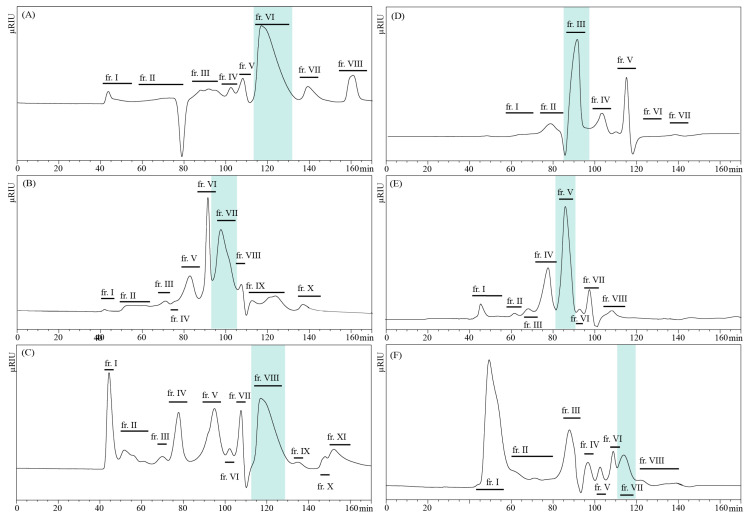
Gel-filtration chromatography profiles of the heteropolysaccharides isolated from LPS of the *Bordetellae* and separated on the Superdex 30 pg, with RI detector. (**A**) *B. pertussis* 606, (**B**) *B. pertussis* 186, (**C**) *B. holmesii* ATCC 51541, (**D**) *B. bronchiseptica* 530, (**E**) *B. bronchiseptica* 1943, and (**F**) *B. parapertussis* 529. The marked regions represent the main fractions used for the structural analysis.

The obtained LPS preparations were hydrolyzed with 1.5% acetic acid. Insoluble lipid A sediments were separated by centrifugation and the soluble heteropolysaccharide reaction products were further separated by size exclusion chromatography using a HiLoad 16/600 Superdex 30 prep grade column equilibrated with 0.05 M acetic acid. All recovered fractions were checked by 1D ^1^H NMR spectroscopy. The largest fraction with the longest retention times corresponding to OS components were recognized as the major core OS for respective *Bordetellae* LPS. The retention times for these main fractions differed. The presence of the fast eluting O-specific polysaccharide (O-PS) fractions in the chromatographic profiles (Figure 2) confirmed the smooth-type LPS of *B. parapertussis* 529 and *B. holmesii* ATCC 51541.

Comparative analyses of heteropolysaccharides and oligosaccharides isolated from the LOS and LPS were performed by NMR spectroscopy techniques. The analyses were conducted under mild conditions to preserve the native structure of the isolated glycans and to minimize the risk of structural changes in preparation, but nonetheless acidic conditions formed various Kdo variants as we previously identified and described [24].

### 2.2. ^1^H NMR Structure-Reporter Groups Analysis of the Bordetellae Core Oligosaccharides

**Figure 3:**
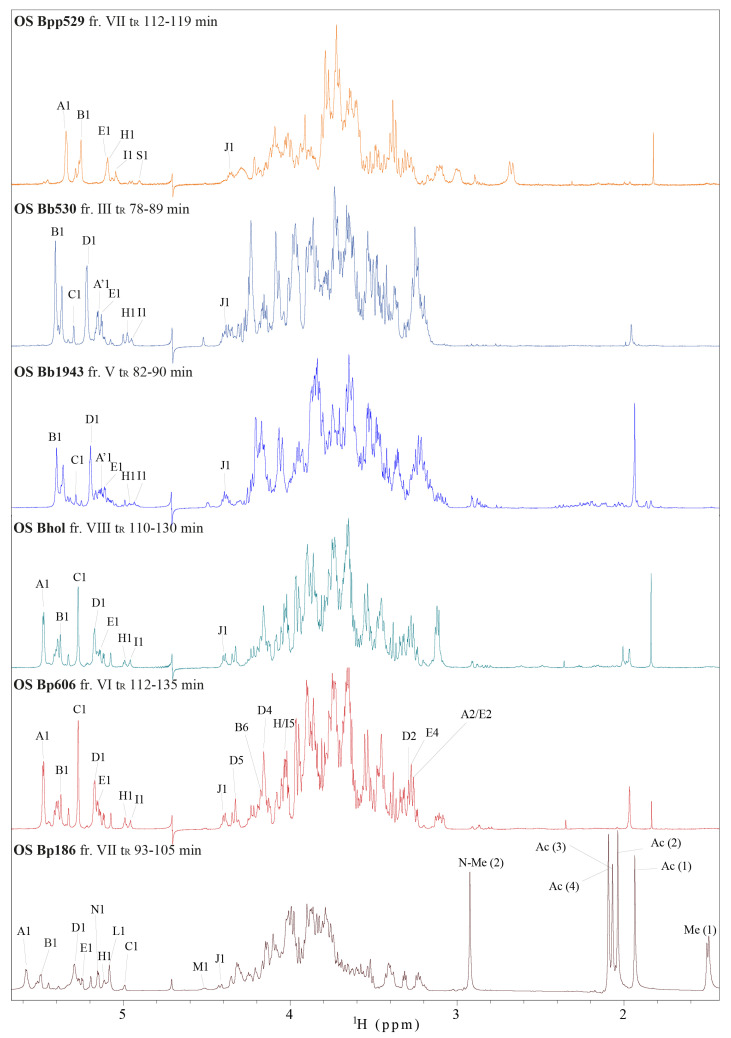
Structure-reporter groups in the ^1^H NMR spectra of the *Bordetellae* oligosaccharides: *B. pertussis* strain 186, *B. pertussis* strain 606, *B. holmesii* ATCC 51541, *B. bronchiseptica* PCM 1943, *B. bronchiseptica* PCM 530, and *B. parapertussis* PCM 529. The capital letters and numbers indicate protons in respective monosaccharides. The resolved resonances in the anomeric regions as well as the characteristic signals of the trisaccharide in the structure of *B. pertussis* 186 lipooligosaccharide (LOS) are indicated. Me(1), an exocyclic CH_3_, N-Me(2), N-methyl at C-4, and Ac(4), N-acetyl of residue **M**; Ac(1) and Ac(3), N-acetyls of the residue **L** at C-2 and C-3, respectively; Ac(2), N-acetyl of residue **N**. The bold was used to increase visibility of the indicated residues throughout the text.

The size-exclusion chromatography data for all the investigated *Bordetella* species and strains suggested some structural OS-related variability. ^1^H NMR spectra of the *Bordetellae* OS (Figure 3) provide information on the reporter groups and constitute spectral patterns that are characteristic and unique for these oligosaccharides. ^1^H NMR profiles of the corresponding OS fractions indicated features that were similar across some of the oligosaccharides, and most notably there were similarities between the OS of *B. pertussis* 606 and *B. holmesii* ATCC 51541 and between *B. bronchiseptica* strains 530 and 1943. The altered positions of some anomeric signals, as well as the presence of acetyl and methyl group resonances in the ^1^H spectrum of *B. pertussis* strain 186 was indicative of a trisaccharide substituting the core OS. It is worth noting that the ^1^H NMR profile of the *B. parapertussis* 529 core OS differed substantially from the other profiles, but this data alone was insufficient to deduce the source of the observed variations. Therefore, the analyses were extended with a set of 2D ^1^H,^13^C- and ^1^H,^1^H-correlation experiments to gain insight into the structural features of the isolated core oligosaccharides.

### 2.3. Structural Analysis of the Core OS

As the ^1^H NMR spectra were complex and did not provide sufficiently unique structure-reporter groups information, the specific data on minor variations could not be deciphered. Thus, to trace the structural similarities and differences across all the analyzed core oligosaccharides, the corresponding spin systems of all the investigated core OS were identified and assigned by two dimensional NMR experiments, including ^1^H, ^1^H COSY; ^1^H, ^1^H TOCSY; ^1^H, ^13^C HSQC-DEPT; ^1^H, ^13^C HMBC; ^1^H, ^1^H NOESY, and ^1^H, ^13^C HSQC-TOCSY. The chemical shift values for monosaccharide constituents of the core oligosaccharides were compiled and compared.

**Figure 4:**
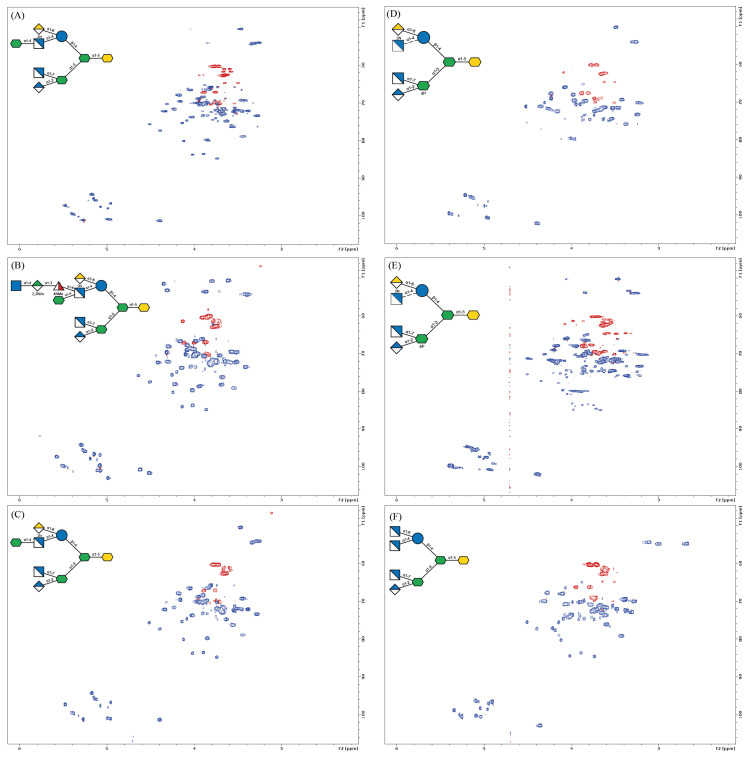
^1^H ^13^C NMR HSQC-DEPT spectra of the oligosaccharides (OS) of *Bordetellae* (**A**) *B. pertussis* 606, (**B**) *B. pertussis* 186, (**C**) *B. holmesii* ATCC 51541, (**D**) *B. bronchiseptica* 530, (**E**) *B. bronchiseptica* 1943, and (**F**) *B. parapertussis* 529 and the symbolic representations of the deduced structures (insets). For the assigned versions of the spectra, refer to the Supplementary Materials, Figures S2–S7.

The ^1^H, ^13^C HSQC-DEPT spectra (Figure 4) present the main regions of interest, depicting the characteristic groups of resonances that correspond to the deduced structures. A number of sugar residues were identified based on the count of anomeric proton and carbon signals, and the presence of aminosugars was indicated by the resonances of amine-bearing carbon signals. The introduced ^13^C-dimension allowed for an unambiguous identification and assignment of all monosaccharide constituents of the oligosaccharides. The identified monosaccharides are denoted with the uppercase letters through the entire text. The assigned versions of the HSQC-DEPT spectra for each OS are included in the Supplementary Materials, Figures S2–S7. The chemical shift values of all individual sugar residues in the same environment were compared and their variability across all the analyzed OS has been presented (Table 1). The monosaccharide residues among different OS were considered as present in the same environment when the types of residues involved and the linkages between all neighboring segments were the same.

Here, we describe the chemical shifts as their mean values and standard deviations of the sample for the individual spin systems of the monosaccharide residues among the core OS of the investigated *Bordetellae*, indicating the identified deviations from a scheme of the complete nonasaccharide core as described for *B. pertussis* 606 [24].

Residue A (δ_H1_/δ_C1_ 5.42/97.8 ppm) was identified as the 4-substituted α-Glc*p*N [→4)-α-Glc*p*N-(1→] based on large coupling constants between H-2, H-3, H-4, and H-5 in the spin system, as well as the relatively high value of the chemical shift of the C-4 (δ_C_ 73.7 ppm) signal. The chemical shift of the C-2 signal (δ_C_ 55.3 ppm) implied substitution with an amine group. The 4,6-disubstituted variant of this residue was identified in the *B. pertussis* strain 186 (residue A*) as a branching point linked to an additional trisaccharide. In the OS of *B. bronchiseptica* strains 530 and 1943, this residue was present in unsubstituted forms assigned as **A’^Bb530^** (δ_H1_/δ_C1_ 5.23/96.2 ppm) and **A’^Bb1943^** (δ_H1_/δ_C1_ 5.22/96.1 ppm). Both residues were recognized as the terminal α‑Glc*p*N [α-Glc*p*N-(1→] based on the large coupling constants between H-2, H-3, H-4, and H-5 in the spin system and the chemical shift of the C-2 signal (δ_C_ **^Bb530^** 55.1 ppm and δ_C_ **^Bb1943^** 55.0 ppm).

Residue B (δ_H1_/δ_C1_ 5.45/100.8 ppm) was recognized as the 2,7-disubstituted-l-*glycero*-α-d-*manno*-Hep*p* [→2,7-l-α-d-Hep*p*-(1→] from the ^1^H and ^13^C chemical shifts, the small vicinal couplings between H-1, H-2, and H-3, and the relatively high chemical shifts of the C-2 (δ_C_ 80.3 ppm) and C-7 (δ_C_ 70.7 ppm) signals.

Residue C (δ_H1_/δ_C1_ 5.27/101.2 ppm) was assigned as the terminal l-*glycero*-α-d-*manno*-Hep*p* [l-α-d-Hep*p*-(1→] based on the ^1^H and ^13^C chemical shifts and the small vicinal couplings between H-1, H-2, and H-3.

Residue **D** (δ_H1_/δ_C1_ 5.26/95.5 ppm) was identified as the terminal α-Gal*p*NA [α-Gal*p*NA-(1→] based on the large couplings between vicinal protons H-1, H-2, and H-3 and the small couplings among H‑3, H-4, and H5. The low chemical shift of the C-2 signal (δ_C_ 51.3 ppm) indicated substitution with an amine group. The characteristic five-proton spin system and the high values of the chemical shifts of H-4 (δ_H_ 4.27 ppm), H-5 (δ_H_ 4.48 ppm), and C-5 (δ_C_ 175.1 ppm) resonances allowed us to identify this residue as aminouronic acid.

Residue **E** (δ_H1_/δ_C1_ 5.20/96.8 ppm) was recognized as the terminal α‑Glc*p*N [α-Glc*p*N-(1→] based on the large coupling constants between H-2, H-3, H-4, and H-5 in the spin system and the chemical shift of the C-2 signal (δ_C_ 55.1 ppm).

Residue **F** (δ_H1_/δ_C1_ 5.15/98.7 ppm) was characterized as the 3,4-disubstituted-l-*glycero*-α-d-*manno*-Hep*p* [→3,4-l-α-d-Hep*p*-(1→] from the ^1^H and ^13^C chemical shifts, the small vicinal couplings between H-1, H-2, and H-3, and the high chemical shifts of the C-3 (δ_C_ 79.9 ppm) and C-4 (δ_C_ 73.4 ppm) signals.

Residues **H** (δ_H1_/δ_C1_ 5.07/100.8 ppm) and **I** (δ_H1_/δ_C1_ 5.04/101.9 ppm) were recognized as α-Glc*p*A [α-Glc*p*A-(1→] based on the five-proton spin systems, the large chemical shifts of H-4 (δ_H_ 3.49 ppm), H-5 (δ_H_ 4.18 ppm), and C-5 (δ_C_ 175.3 ppm) signals for residue **H**, the chemical shifts of H-4 (δ_H_ 3.49 ppm), H-5 (δ_H_ 4.17 ppm), and C-5 (δ_C_ 176.6 ppm) for residue **I**, and large couplings among H-2, H-3, H-4, and H-5.

Residue **J** (δ_H1_/δ_C1_ 4.47/102.9 ppm) was assigned as the 4,6-disubstituted-β-Glc*p* [→4,6-β-Glc*p*-(1→] based on the large couplings among all protons in the spin system and the relatively high value of the chemical shift of the C-4 (δ_C_ 78.5 ppm) and C-6 (δ_C_ 68.1 ppm) carbon signals.

Residue **K** (δ_H1_/δ_C1_ 4.98/97.5 ppm) was recognized as the terminal α‑Glc*p*N [α-Glc*p*N-(1→] based on the large coupling constants between H-2, H-3, H-4, and H-5 in the spin system and the chemical shift of the C-2 signal (δ_C_ 55.8 ppm). This residue was only present in the OS of *B. parapertussis* 529. It was linked to residue **J** at position C-6 as confirmed by an HMBC connectivity observed between the C-1 of residue **K** (δ_C1_ 97.5 ppm) and H-6,6′ (δ_H_/δ_C_ 3.88, 3.95 ppm) of residue **J**. The residue **K** effectively replaced the residue **D** identified among all other *Bordetella* core OS.

In all the core OS 2D ^1^H,^13^C HSQC-DEPT spectra Kdo residues were present in two forms of 4,7-anhydro-3-deoxy-d-*manno*-2-octulofuranose acid instead of 2-keto-3-deoxy-d-*manno*-octulosonic acid (Kdo*f* and Kdo*f*’) [25,26]. This observation can be explained by the acidic conditions used for LPS and OS isolation leading to β-elimination of phosphate from the C-4 position of Kdo in the native OS [27]. The sequence of sugar residues was confirmed for each of the respective *Bordetellae* core oligosaccharides by inter-residue cross-peaks between the transglycosidic protons observed in the NOESY experiments and between the anomeric protons and carbons at the linkage position in the HMBC spectra. The complete assignment of the ^1^H and ^13^C chemical shift data for the investigated core oligosaccharides have been included as a set of annotated HSQC-DEPT spectra in the Supplementary Materials Figures S2–S7 [24]. The observed chemical shift patterns of all the monosaccharide constituents of the core OS (Supplementary Materials Figure S1) were consistent with those described previously [28].

### 2.4. Presence of Phosphate Groups as an Additional Source of Variation among Bordetellae Core Oligosaccharides

**Figure 5:**
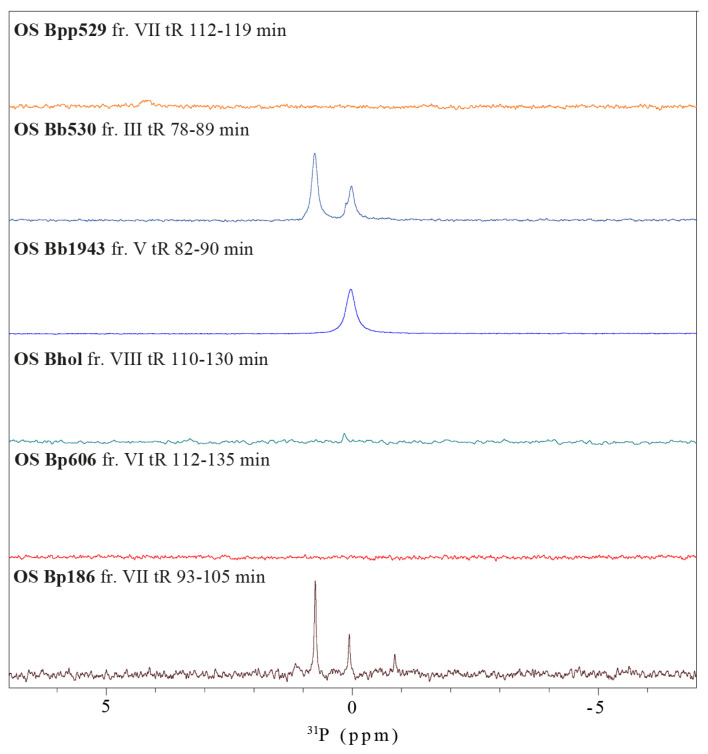
^31^P NMR spectra of the main core oligosaccharides of *Bordetellae* LPS.

To further describe the minor variations between the core oligosaccharides with similar retention times in the chromatographic profiles of heteropolysaccharides, the main OS fractions were analyzed for the presence of phosphate groups using 1D ^31^P NMR spectroscopy (Figure 5). Subsequently, whenever the presence of phosphates was detected, ^1^H, ^31^P correlation spectra were recorded to identify the substituted positions in the OS.

The 1D ^31^P NMR spectra indicated the presence of phosphate groups in the core OS of *B. bronchiseptica* strains 530 and 1943, as well as in *B. pertussis* 186. The phosphate groups substituted the residue **B** (2,7-l-α-d-Hep*p*) at the C-4 position. In the ^31^P NMR spectra of *B. pertussis* 606, *B. holmesii* ATCC51541, and *B. parapertussis* 529, signals of phosphate groups were not detected. However, the identified spin systems of 4,7-anhKdo*f* and 4,7-anhKdo*f*’ in all the core OS imply the substitution of Kdo by phosphate groups in native OS.

**Figure 6:**
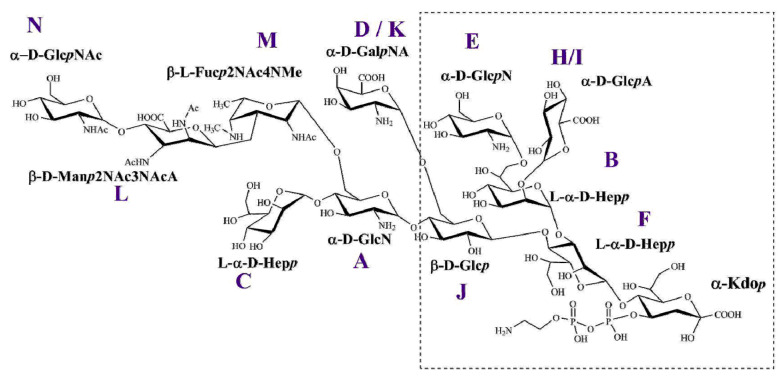
The conserved structural elements among the core oligosaccharides of *Bordetellae*. The uppercase letters refer to carbohydrate residues. Residues **L**, **M**, and **N** constitute the trisaccharide identified in the *B. pertussis* 186 LOS. Residues **A** and **C** in the LPS of *B. bronchiseptica* were either not detected or identified at much lower contour levels. Residue **D** was identified among all LPS of *Bordetellae*, except for in *B. parapertussis* 529, in which it was replaced by residue **K**. The identified “lowest common structural denominator” has been framed with a dashed line.

The combined NMR data allowed for the identification of the least variable segment, conserved among all the investigated *Bordetellae*, and indicated core-related sources of structural variation. The innermost hexasaccharide, comprising the residues **E**, **H**/**I**, **J**, **B**, **F**, and the Kdo, is the “lowest common denominator” structure conserved among all the investigated core oligosaccharides of *Bordetellae*. (Figure 6).

## 3. Discussion

The complete structural data on any given hypothetical carbohydrate antigen is a prerequisite for further analysis of structure-related activities and interactions with hosts triggered by these molecules. The structural features of the LPS isolated from various species and strains of genus *Bordetella* have been broadly covered in the relevant literature. The LPS of *B. pertussis* structurally form lipooligosaccharides (LOS) as they lack the O-specific polysaccharide, which is replaced by a distal trisaccharide [12]. Other species from the genus *Bordetella* have more structurally diverse LPS. The most extensive research concerning the LPS structures of *B. bronchiseptica* and *B. parapertussis* was reported by Preston et al. The O-antigen for classical *Bordetellae* was defined as a 2,3-diacetamido-α-l-2,3-dideoxygalactouronic acid homopolymer [29] with three types of O-PS [13,30,31]. In the analyses of the heteropolysaccharides of *B. parapertussis* and *B. bronchiseptica*, an additional pentasaccharide fragment linking the O-specific polysaccharide to the core OS was also identified. Typical *B. bronchiseptica* core OS were found to be dodecasaccharides containing the distal trisaccharide. The oligosaccharide core of *B. parapertussis* is partially devoid of α-d-Gal*p*NA, terminal l-α-d-Hep*p,* and the distal trisaccharide is completely absent [13].

The results we obtained from the analysis of *B. pertussis* LPS isolated from strains 186 and 606 were in agreement with the previous reports regarding the overall scheme of these lipooligosaccharides and the structure-reporter groups of the dodecasaccharide of strain 186, i.e., characteristic resonances of →4)-β-d-Man*p*2NAc3NAcA-(1→, →3)-β-l-Fuc*p*2NAc4NMe-(1→ and α-d-Glc*p*NAc-(1→ residues were clearly identifiable. The ^1^H NMR profiles of the LOS-derived oligosaccharides of strains 186 and 606 were used as references in comparisons with other *Bordetella* strains. As we previously reported, the core oligosaccharide of an emerging pertussis-like pathogen *B. holmesii* ATCC 51541 was identical to the core nonasaccharide of *B. pertussis* strain 606 [24].

The analyzed *B. bronchiseptica* strains 530 and 1943 appeared as rough types, devoid of O-specific polysaccharides. As we demonstrated previously, LPS of *B. bronchiseptica* 530 and 1943 weakly cross-reacted in serological analyses with serum containing polyclonal antibodies directed against the core OS, suggesting the incomplete oligosaccharide in its structure [24]. The comparison of the ^1^H, ^13^C HSQC-DEPT spectra of the main *B. bronchiseptica* OS fractions to the known core structures of the *B. pertussis* strains 186 and 606, showed that the minimal *B. bronchiseptica* core OS is a heptasaccharide devoid of α-d-Glc*p*N (residue **A**) and terminal α-d-Hep*p* (residue **C**). This observation also explains the lack of distal trisaccharide in the analyzed structures in the main core OS fraction, as the residue **A** is a monosaccharide typically substituted with a linker or the trisaccharide. These observations are contrary to the original claim by Preston at al. that the *B. bronchiseptica* core is identical to the *B. pertussis* dodecasaccharide. Our comparison of *B. bronchiseptica* main core OS fractions with those of other *Bordetellae* indicates that the core identity is limited to the innermost hexassacharide and α-d-Gal*p*NA (residue **D**) as the conserved elements. The trisaccharide segment, as well as l-α-d-Hep*p* (residues **C**) and α-d-Glc*p*N (residue **A**), are absent or represented only in minor fractions.

The NMR data both from the ^1^H NMR profiles and 2D HSQC-DEPT spectra of the *B. parapertussis* 529 heteropolysaccharide indicated structural features different from those observed for other investigated core OS, but also different from the literature data on LPS of *B. parapertussis* [13]. Most notably, in the main OS fraction (Figure 2F; fr. VII), no α-d-Gal*p*NA (residue **D**) was identified, but instead the residue J was substituted at the C-6 position by α-Glc*p*N (residue **K**). This was confirmed by the HMBC connectivity observed between the C-1 of residue **K** and H-6, 6′ of residue **J**. The residue **A** appeared as the terminal α-d-Glc*p*N and the l-α- d-Hep*p* (residue **C**) was missing in the main OS fraction. However, in the chromatographic profile, a fraction with a shorter retention time and matching that of the *B. pertussis* 186 dodecasaccharide was spotted. In the NMR spectra of this fraction (fr. III), the terminal l-α-d-Hep*p*, α-d-Gal*p*NA, and weak signals from the distal trisaccharide were identified.

We conclude that the innermost hexasaccharide, comprising the α‑Glc*p*N, α-Glc*p*A, 4,6-disubstituted-β-Glc*p*, 2,7-disubstituted-l-α-d-Hep*p*, 3,4-disubstituted-l-α-d-Hep*p* residues, and the Kdo, is the least variable OS segment conserved among all the investigated core oligosaccharides of *Bordetellae.* This minimal common element in the structure of *Bordetellae* lipopolysaccharides could be used to prepare a universal cross-protective vaccine component against infections with various species of bacteria of the genus *Bordetella*. The generated antibodies could be capable of binding to the conserved *Bordetella* OS in a similar manner that the antibodies against OS of rough *E. coli* strains cross-recognize the core oligosaccharides among smooth strains [22].

Our understanding of the possible role of discrete structural elements that are involved in the interactions with specific antibodies is vital for the design of new, safer, and more effective neoglycoconjugate vaccines. The use of anti-neoglycoconjugate antibodies combined with NMR-intensive techniques seems especially useful in the identification of target glycans and mapping of bacterial surface epitopes at the molecular level. Such data can be subsequently correlated with the complementary immunological analysis of in vitro anti-endotoxin properties of the antibodies, allowing for a better selection of defined glycans as immunogens.

## 4. Materials and Methods

### 4.1. Bacteria

*B. pertussis* strains 186 and 606, used in the current wP vaccine manufactured in Poland, came from the National Medicines Institute (Warsaw, Poland) [32]. *Bordetella parapertussis* PCM 529 (ATCC 15311), *Bordetella bronchiseptica* PCM 530 (ATCC 19395), and *Bordetella bronchiseptica* PCM 1943 (ATCC 4617) were acquired from the PCM collection (Hirszfeld Institute of Immunology and Experimental Therapy, Polish Academy of Sciences, Wrocław, Poland). *Bordetella holmesii* strain ATCC 51541 (DSM 13416) was obtained from the DSMZ collection (Leibniz Institute, Braunschweig, Germany). The strains were stored as bacterial suspensions in PBS containing 20% glycerol, at −70 °C. Bacteria were grown on charcoal agar medium supplemented with 10% defibrinated sheep blood (GRASO Biotech, Starogard Gdański, Poland) and then transferred to the liquid medium. *B. pertussis, B. parapertussis, B. holmesii,* and *B. bronchiseptica* strains were cultured using Stainer–Scholte medium at 37 °C for 72 h. Bacteria were killed with 1% phenol, harvested by centrifugation (4000× *g*, 30 min, 4 °C) (Sorvall Lynx 6000), suspended in water, and freeze-dried.

### 4.2. Lipopolysaccharides and Heteropolysacharide Fractions

LPS was extracted from lyophilized bacterial cells by the modified hot phenol/water extraction method [23] and purified by ultracentrifugation [33]. The modified extraction included an extra step prior to the addition of phenol. Briefly, killed and lyophilized bacteria were suspended in 0.05 M phosphate buffer at pH 7.4 and lysozyme (EC 3.2.1.17, specific activity ≥ 40,000 U/mg) was added in portions (10 mg per one g of dry bacterial mass) and the suspension was incubated for 18 h at 25 °C with stirring [34]. LPS (45 mg) was hydrolyzed with 1.5% acetic acid at 100 °C for 15 min and subsequently water-soluble heteropolysaccharides were isolated. The amounts of the heteropolysaccharides recovered varied between the species and strains, ranging between 10 and 22 mg. The supernatant was fractionated using the semi-preparative HPLC UltiMate 3000 chromatographic system (Dionex Corporation, Sunnyvale, CA, USA) on a HiLoad 16/600 Superdex 30 prep grade column (30 mm × 124 cm, grain size 34 μm, GE Healthcare, Chicago, IL, USA) equilibrated with 0.05 M acetic acid. Eluates were monitored with a Shodex RI-102 detector (Showa-Denko, Tokio, Japan). The chromatography yielded the main fractions containing O-specific polysaccharides (if present), separated from shorter chains, and the core oligosaccharides. All fractions were checked by NMR spectroscopy. The main OS fractions were selected for further NMR analysis, providing a complete description of the monosaccharide components by a set of 2D ^1^H,^13^C- and ^1^H,^1^H-correlation experiments.

### 4.3. SDS-PAGE

The LPS was analyzed by SDS-PAGE according to the method of Laemmli [34]. The LPS bands were visualized by the silver staining method [35].

### 4.4. NMR Spectroscopy

NMR spectra of the isolated oligosaccharides were recorded for ^2^H_2_O solutions at 25 °C on a Bruker Avance III 600 MHz spectrometer (Bruker Biospin GmbH, Rheinstetten, Germany) using 5 mm QCI cryoprobe; 3 mm tubes (~160 μL) were used for the measurements. Oligosaccharide fractions were repeatedly exchanged with ^2^H_2_O with intermediate lyophilization. Acetone (δ_H_/δ_C_ 2.225/31.05 ppm) was used as an internal reference. The data were acquired and processed using TopSpin software (Bruker BioSpin GmbH, Rheinstetten, Germany). The NMRFAM-SPARKY program (NMRFAM University of Wisconsin-Madison, Madison, Wisconsin, USA) was used for assignments of all processed spectra [36]. The signals were identified by one- and two-dimensional experiments (COSY, TOCSY, NOESY, HMBC, HSQC-DEPT, and HSQC-TOCSY). In the TOCSY experiments, the mixing times used were 30, 60, and 100 ms. The coupling patterns within the identified spin-systems in the 2D TOCSY experiments facilitated the identification of individual monosaccharide residues. The delay time in the HMBC experiment was 60 ms and the mixing time in the NOESY experiment was 100 ms.

## 5. Conclusions

The 1D ^1^H NMR profiles combined with 2D ^1^H,^13^C HSQC-spectra can be a useful tool in the identification of minute structural features among LPS of *Bordetellae*. It allowed for an immediate identification of structural variations among the core OS of *B. bronchiseptica* strains 530 and 1943 and *B. parapertussis* strain 529.

The innermost hexasaccharide, comprising the α‑Glc*p*N, α-Glc*p*A, 4,6-disubstituted-β-Glc*p*, 2,7-disubstituted-l-α-d-Hep*p*, 3,4-disubstituted-l-α-d-Hep*p* residues, and the Kdo, was identified as the least variable OS segment, conserved among all the investigated core oligosaccharides of *Bordetellae*.

The identified “lowest common structural denominator” concept could be employed to facilitate design of a cross-reactive carbohydrate segment of the glycoconjugate vaccines.

## Figures and Tables

**Table 1:** ^1^H,^13^C chemical shifts of monosaccharide OS components analyzed in *Bordetellae* in the same environment **^#^.**

Residue	Chemical Shifts (ppm)							
	H-1	H-2	H-3	H-4	H-5	H-6, H-6′	H-7	H-8, H-8′
	C-1	C-2	C-3	C-4	C-5	C-6	C-7	C-8
**Kdo** 4,7-anhKdo*f*			3.19	4.55	4.17	4.18	3.87	3.74, 3.67
		203.7	43.7	78.3	84.7	76.4	84.6	62.0
		(0.695)	(0.042)	(0.083)	(0.434)	(0.129)	(0.073)	(0.047)
**Kdo**’ 4,7-anhKdo*f*			3.18	4.59	4.20	4.16	3.82	3.74
		203.8	39.9	76.6	81.0	75.6	85.6	62.7
		(0.768)	(0.106)	(0.101)	(0.254)	(0.071)	(0.066)	(0.051)
**A** 4,(6*)-α-d-Glc*p*N	5.42	3.33	3.93	3.59	3.80	3.92, 3.80		
	(0.157)	(0.125)	(0.108)	(0.114)	(0.085)	(0.099, 0.008)		
	97.8	55.3	71.3	73.7	73.0	62.3 *		
	(1.410)	(0.264)	(1.428)	(3.807)	(1.073)	(2.717)		
**B** 2,7-l-α-d-Hep*p*	5.45	4.05	4.02	4.13 ^	3.62	4.29	3.80	
	(0.053)	(0.136)	(0.064)	(0.241)	(0.088)	(0.041)	(0.016)	
	100.8	80.3	71.3	69.2	72.8	68.8	70.7	
	(0.283)	(1.099)	(0.398)	(2.056)	(0.362)	(0.090)	(0.585)	
**C**l-α- d-Hep*p*	5.27	4.04	3.72	3.90	3.71	4.03	3.70	
	(0.197)	(0.007)	(0.114)	(0.043)	(0.087)	(0.043)	(0.018)	
	101.2	70.2	71.2	65.6	72.2	68.3	62.5	
	(1.124)	(0.225)	(1.131)	(0.366)	(0.291)	(0.174)	(0.104)	
**D** α-d-Gal*p*NA	5.26	3.55	4.13	4.27	4.48			
	(0.025)	(0.016)	(0.02)	(0.036)	(0.106)			
	95.5	51.3	67.4	70.4	72.8	175.1		
	(0.27)	(0.108)	(0.303)	(0.299)	(0.539)	(1.034)		
**E** α-d-Glc*p*N	5.20	3.32	3.92	3.51	3.82	3.80, 3.86		
	(0.012)	(0.063)	(0.03)	(0.018)	(0.134)	(0.004, 0.015)		
	96.8	55.1	70.9	70.3	73.3	61.1		
	(0.319)	(0.111)	(0.451)	(0.084)	(0.646)	(0.063)		
**F** 3,4-l-α- d-Hep*p*	5.15	3.98	4.03	4.25	3.65	4.06	3.75	
	(0.010)	(0.018)	(0.129)	(0.07)	(0.062)	(0.062)	(0.01)	
	98.7	74.2	79.9	73.4	72.3	69.4	63.6	
	(0.308)	(1.553)	(1.392)	(0.988)	(0.053)	(0.430)	(0.308)	
**H** α-d-Glc*p*A	5.07	3.59	3.78	3.49	4.18			
	(0.055)	(0.018)	(0.032)	(0.045)	(0.124)			
	100.8	71.8	72.1	72.1	73.1	175.3		
	(0.266)	(0.214)	(0.597)	(0.417)	(1.016)	(1.72)		
**I** α-d-Glc*p*A	5.04	3.59	3.77	3.49	4.17			
	(0.041)	(0.018)	(0.035)	(0.044)	(0.116)			
	101.9	72.9	72.9	73.0	74.1	176.6		
	(0.242)	(0.207)	(0.551)	(0.352)	(1.009)	(1.382)		
**J** 4,6-β-d-Glc*p*	4.47	3.35	3.58	3.54	3.60	3.88, 3.95		
	(0.014)	(0.031)	(0.104)	(0.056)	(0.236)	(0.074, 0.038)		
	102.9	74.3	77.1	78.5	72.7	68.1		
	(0.634)	(0.166)	(0.248)	(2.322)	(1.292)	(0.523)		
**L** 4-β-d-Man*p*2NAc3NAcA	5.04	4.30	4.27	3.89	3.94			
	100.6	52.3	54.0	79.4	71.3	175.6		
**M** 3-β-l-Fuc*p*2NAc4NMe	4.57	3.74	4.17	3.75	4.03	1.44		
	101.5	51.6	79.0	63.1	68.6	17.3		
**N** α-d-Glc*p*NAc	5.10	3.81	3.67	3.47	3.70	3.76, 3.81		
	97.5	54.4	71.3	70.4	72.6	60.7		
**A’ Bb530** α-d-Glc*p*N	5.23	3.38	3.94	3.50	3.77	3.78, 3.84		
	96.2	55.1	70.8	70.3	73.1	61.1		
**A’ Bb1943** α-d-Glc*p*N	5.22	3.38	3.92	3.49	3.74	3.77, 3.83		
	96.1	55.0	70.8	70.3	73.1	61.1		
**K Bpp529** α-Glc*p*N	4.98	2.75	3.79	3.36	3.71	3.86, 3.79		
	97.5	55.8	73.5	71.0	72.5	61.4		

^#^ Mean values of the corresponding monosaccharaide residues in the same environment for all analyzed core OS; the absolute standard deviation values for the samples are placed in parentheses; Standard deviation values (were ≤ 0.02) for the ^1^H resonances of Kdo and Kdo’ were omitted. * For *B. pertussis* 186, C6/H6 chemical shift values of residue **A** are different due to the presence of distal trisaccharide (δ_C_ 67.9/δ_H_ 4.06 ppm); ^ For *B. bronchiseptica* 530, C4/H4 chemical shift values of residue **B** differ due to the presence of a phosphate group (Bb 530 δ_C_ 72.1/δ_H_ 4.43 ppm); Residues **L, M**, and **N** constitute the distal trisaccharide of the OS of *B. pertussis* 186; Residue **K** is present in the OS of *B. parapertussis* 529 instead of residue **D**, and residue **A**’ is in the OS of *B. bronchiseptica* strains 530 and 1943. Assignment of residue **C** in the OS of *B. bronchiseptica* strains 530 and 1943 was only tentative as the substitution with this residue was incomplete. The anomeric configuration of the residues was deduced from the J_C1,H1_ couplings provided by the non-decoupled HSQC and HMBC experiments. Residues with the corresponding J_C1,H1_ values (parenthesized) were recognized as α‑pyranosyls: **A** (175 Hz), **B** (178 Hz), **C** (170 Hz), **D** (171 Hz), **E** (173 Hz), **F** (173 Hz), **H**/**I** (170 Hz), **K** (176 Hz), and residue **J** (161 Hz) as β-pyranosyl.

## Data Availability

The data presented in this study have been disclosed in the main text and the Supplementary Materials.

## Supplementary Materials

Supplementary Materials can be found at https://www.mdpi.com/1422-0067/22/3/1029/s1.
