# Supplementary material for: Conserved Structural Features of Core Oligosaccharides among the Lipopolysaccharides of Respiratory Pathogens from the Genus Bordetella Analyzed Exclusively by NMR Spectroscopy
Source: Int J Mol Sci. 2021 Jan 21;22(3):1029. doi: 10.3390/ijms22031029 (PMC7864354; doi:10.3390/ijms22031029)
Supplement: Supplementary file 1 [file ijms-22-01029-s001.pdf]

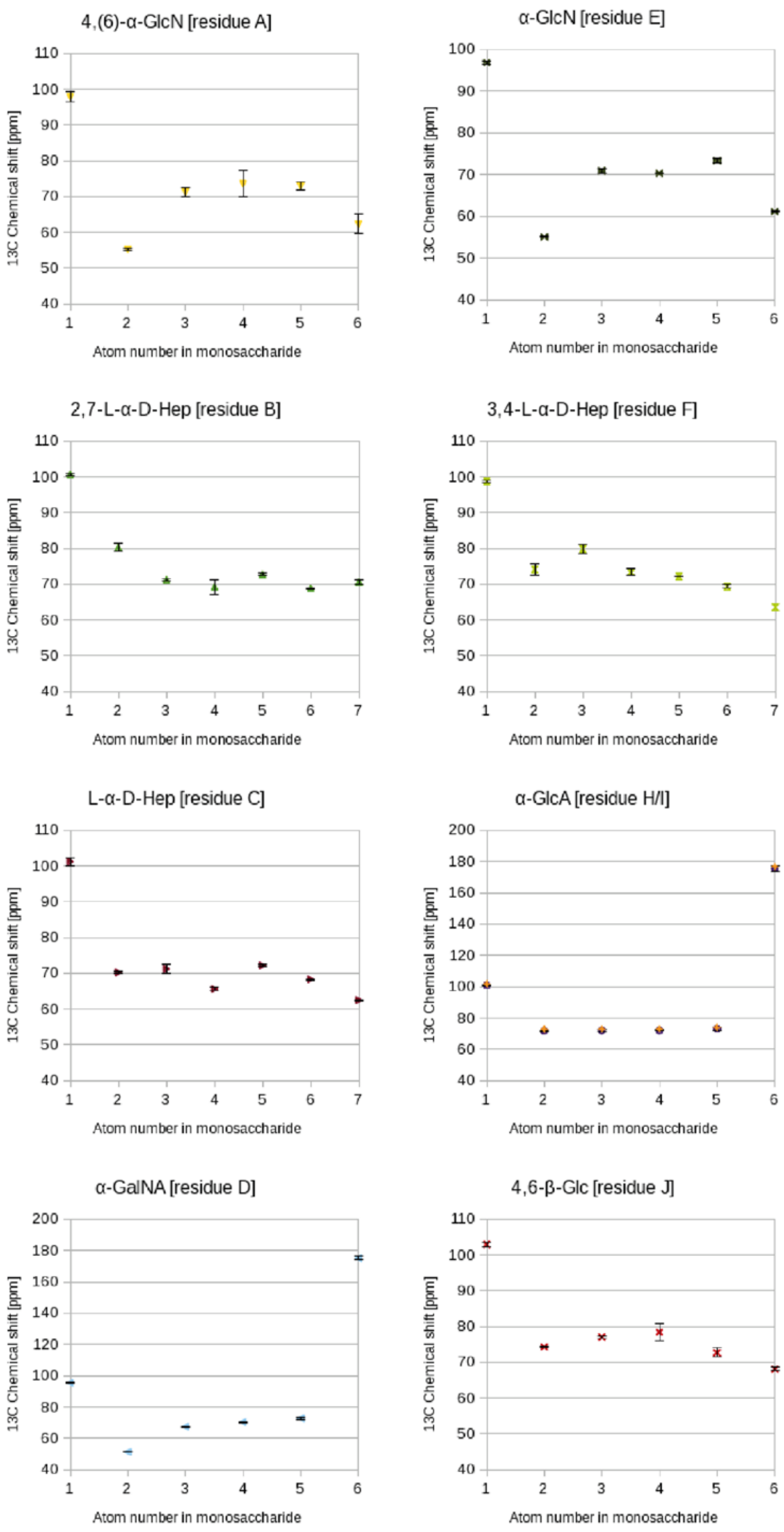

Figure S1. ^{13}C chemical shift patterns of the monosaccharide constituents of the *Bordetellae* core OS. Symbols represent mean and error bars indicate standard deviation values for the monosaccharide residues in the same environment. For some atoms error bars are smaller than the chemical shift marker.

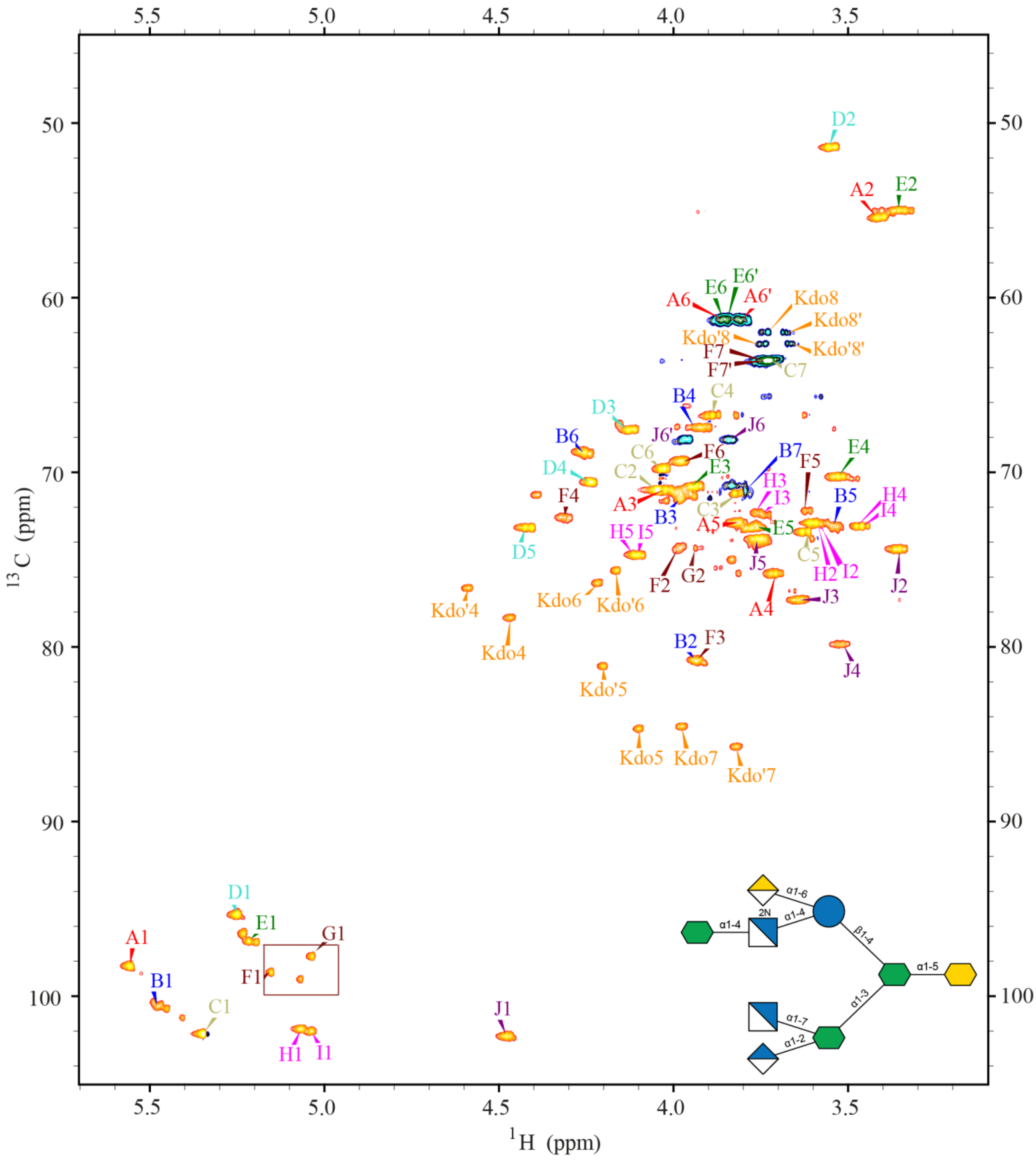

Figure S2. ^1H ^{13}C NMR HSQC-DEPT spectrum of the core OS of *B. pertussis* 606; The spectrum was obtained for the ^2H_2O solutions at 600 MHz and 298K. The cross-peaks are labeled as explained in the text and Table 1. The anomeric resonances F1/G1 (framed) indicate variants of residue F in different environments. The spin systems of these variants were not resolved.

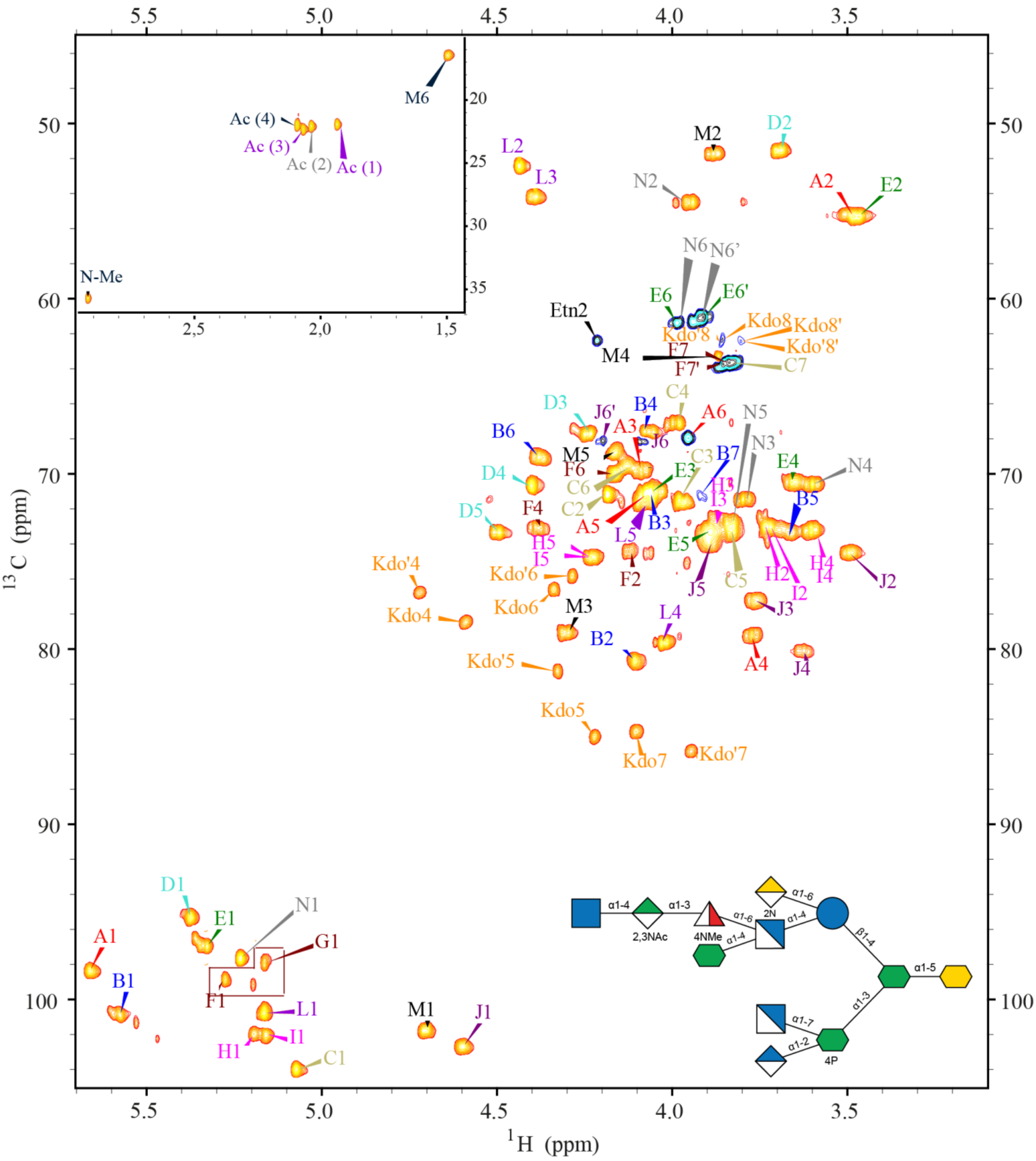

Figure S3. ^{1}H ^{13}C NMR HSQC-DEPT spectrum of the core OS of *B. pertussis* 186; The spectrum was obtained for the $^{2}H_2O$ solutions at 600 MHz and 298K. The cross-peaks are labeled as explained in the text and Table 1. The anomeric resonances F1/G1 (framed) indicate variants of residue F in different environments. The spin systems of these variants were not resolved. The inset shows the characteristic signals of the trisaccharide in the structure of core OS Me, an exocyclic CH_3 (M6), N-Me, N-methyl at C-4, and Ac(4), N-acetyl of residue M; Ac(1) and Ac(3), N-acetyls of the residue L at C-2 and C-3, respectively; Ac(2), N-acetyl of residue N.

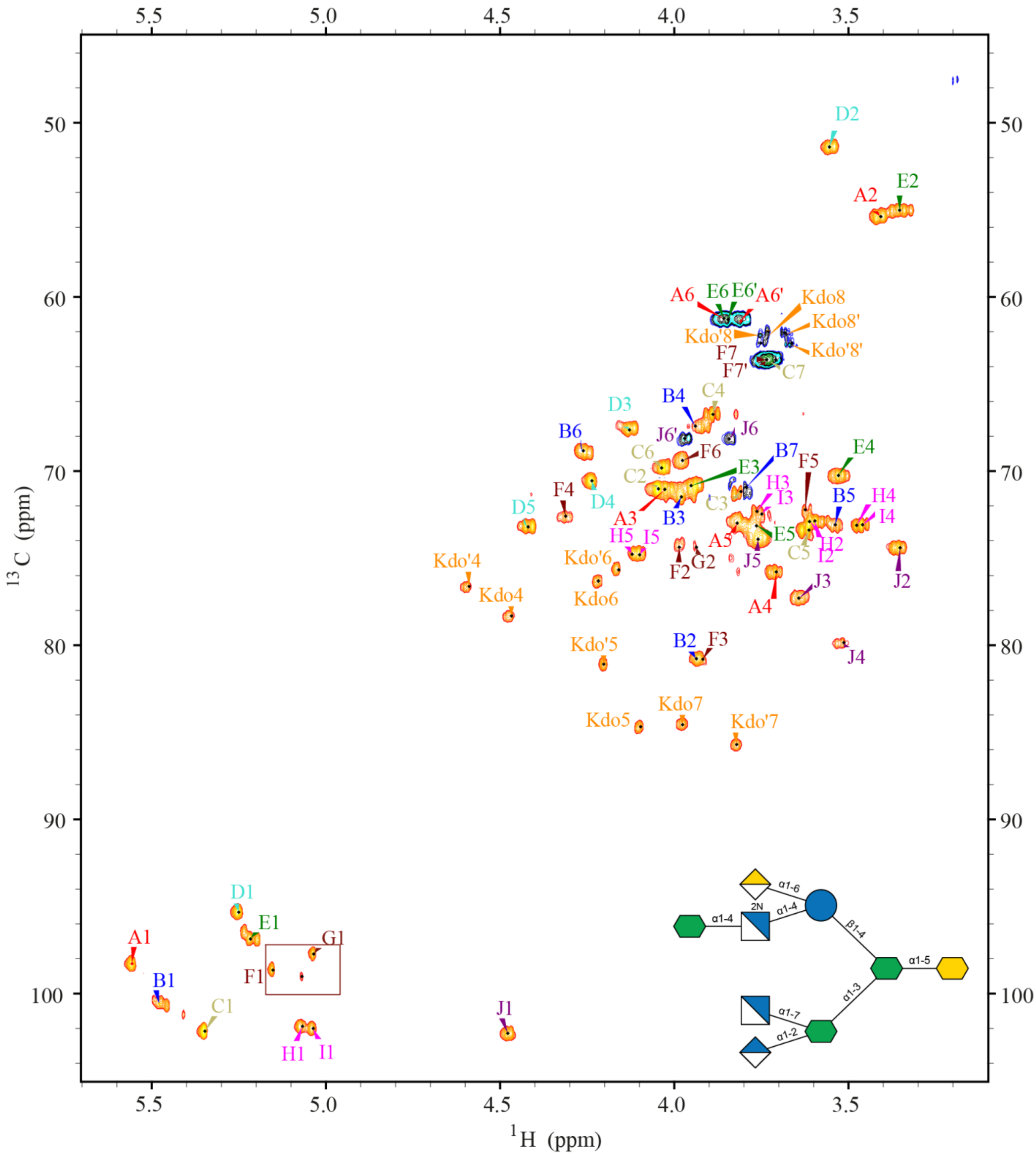

Figure S4. ^{1}H ^{13}C NMR HSQC-DEPT spectrum of the core OS of *B. holmesii* ATCC 51541; The spectrum was obtained for the $^{2}H_2O$ solutions at 600 MHz and 298K. The cross-peaks are labeled as explained in the text and Table 1. The anomeric resonances F1/G1 (framed) indicate variants of residue F in different environments. The spin systems of these variants were not resolved.

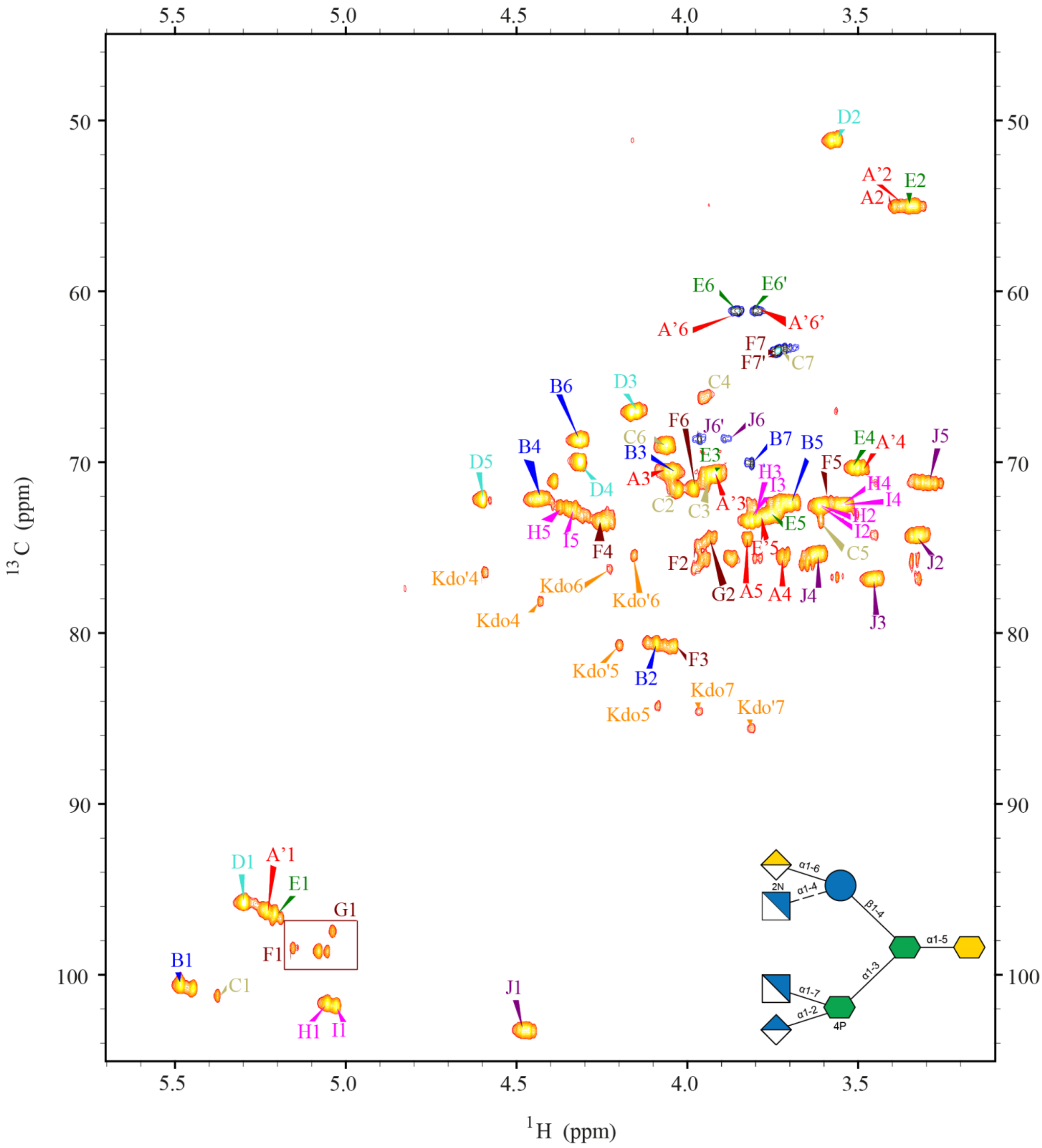

Figure S5. ^{1}H ^{13}C NMR HSQC-DEPT spectrum of the core OS of *B. bronchiseptica* strain 530; The spectrum was obtained for the $^{2}H_2O$ solutions at 600 MHz and 298K. The cross-peaks are labeled as explained in the text and Table 1. The anomeric resonances F1/G1 (framed) indicate variants of residue F in different environments. The spin systems of these variants were not resolved.

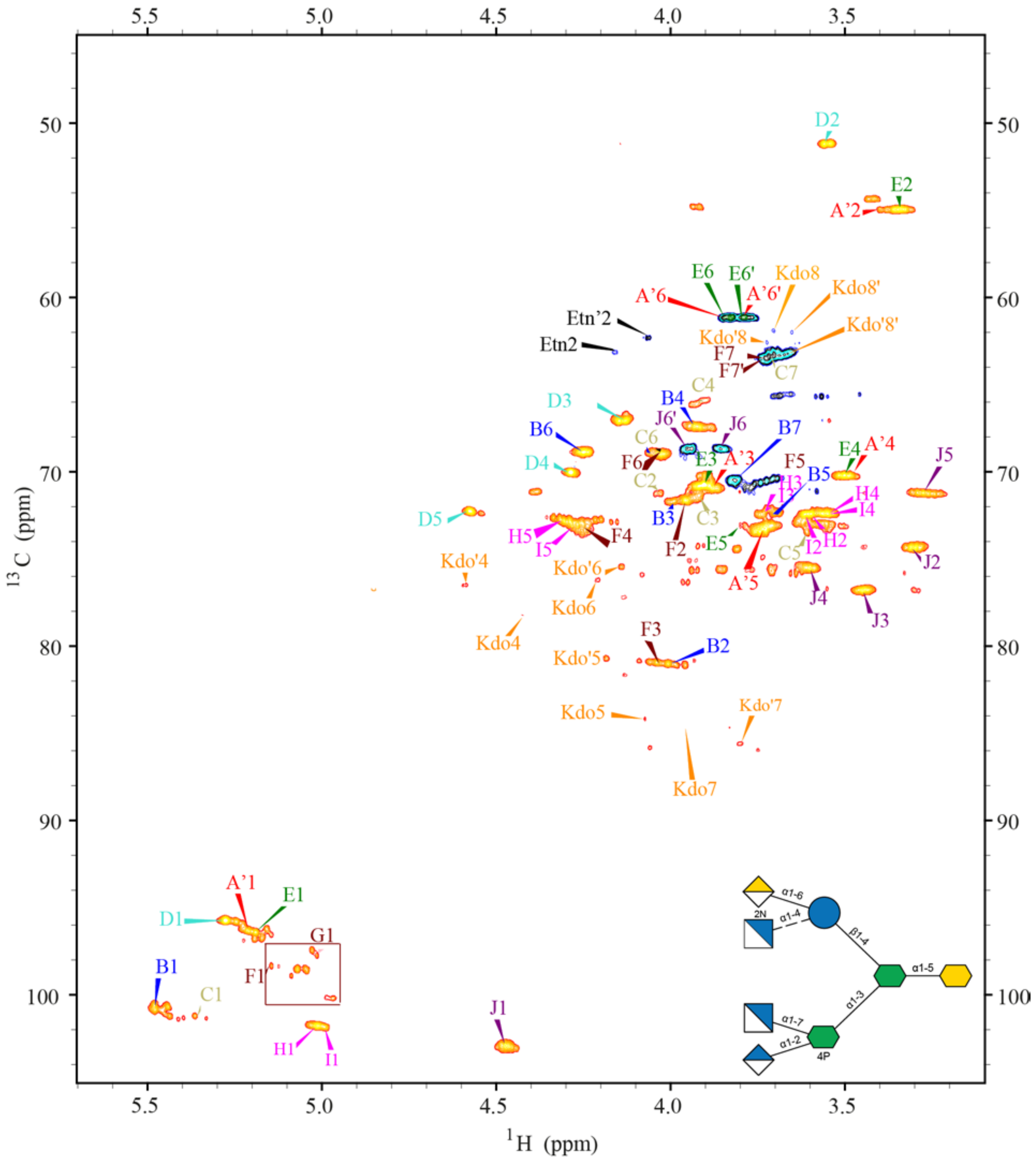

Figure S6. ^{1}H ^{13}C NMR HSQC-DEPT spectrum of the core OS of *B. bronchiseptica* strain 1943; The spectrum was obtained for the $^{2}H_2O$ solutions at 600 MHz and 298K. The cross-peaks are labeled as explained in the text and Table 1. The anomeric resonances F1/G1 (framed) indicate variants of residue F in different environments. The spin systems of these variants were not resolved.

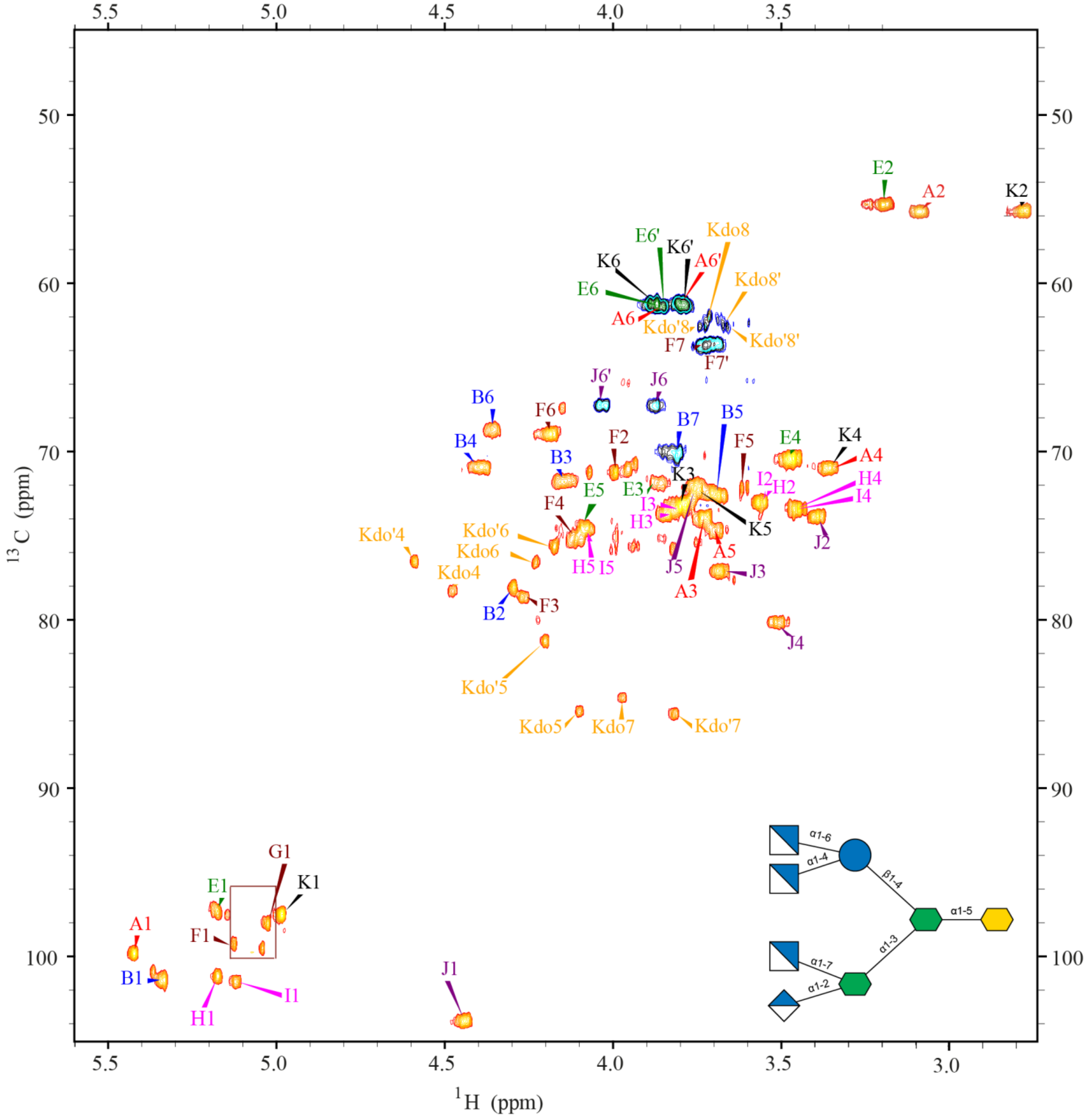

Figure S7. ^{1}H ^{13}C NMR HSQC-DEPT spectrum of the core OS of *B. parapertussis* strain 529; The spectrum was obtained for the $^{2}H_2O$ solutions at 600 MHz and 298K. The cross-peaks are labeled as explained in the text and Table 1. The anomeric resonances F1/G1 (framed) indicate variants of residue F in different environments. The spin systems of these variants were not resolved.
